# Secretory Meningioma of the Right Frontal Lobe: Clinical Presentation and Pathological Insights

**DOI:** 10.7759/cureus.61759

**Published:** 2024-06-05

**Authors:** Daniel Markov, Kristian I Bechev, Vladimir Aleksiev, Ilian Koev

**Affiliations:** 1 General and Clinical Pathology, Medical University of Plovdiv, Plovdiv, BGR; 2 Clinical Pathology, University Hospital Pulmed, Plovdiv, BGR; 3 Neurological Surgery, University Hospital Pulmed, Plovdiv, BGR; 4 Cardiovascular Surgery, Medical University of Plovdiv, Plovdiv, BGR; 5 Neurosurgery, University Hospital Pulmed, Plovdiv, BGR

**Keywords:** brain tumors, frontal lobe of the brain, pathohistological characteristics of meningiomas, convexity meningioma, secretory meningioma

## Abstract

This article presents a case study of a rare convexity meningioma located in the frontal lobe of the right cerebellar hemisphere. Meningiomas comprise a substantial part of central nervous system neoplasms and are classified into benign, atypical, or anaplastic categories, each encompassing a variety of histological subtypes, among which the secretory meningioma is notably rare.

A 77-year-old male presented with a clinical history of headache, impaired memory functions, an initial form of apathetic-abulic syndrome, and a single seizure, which were considered to be indicative of epileptic symptoms that had been present for several weeks. The imaging studies conducted showed a convexity tumor characterized by a rounded morphology and homogeneous contrast enhancement, positioned adjacent to the frontal lobe's cortical surface.

This clinical report details the pathology of a secretory type of meningioma, which is distinguished by the atypical epithelial differentiation of meningothelial cells, resulting in hyaline fiber production. The neoplasm's anatomical accessibility permitted successful surgical resection. The tumor's position was appropriate for surgical removal, and the histological variant, along with the patient's favorable clinical course, is of particular scientific interest.

## Introduction

Secretory meningiomas represent a rare subtype of benign meningiomas and are classified as grade I by the World Health Organization (WHO). Meningiomas represent approximately 36.6% of all tumors of the central nervous system (CNS), with histological preparations indicating that they account for approximately 53.2% of non-malignant CNS tumors [1,2]. According to some clinical studies, secretory meningiomas comprise between 1.1% and 3.0% of all meningiomas [3]. In the majority of scientific studies, a female susceptibility to the disease has been consistently reported. In our case, the affected patient was a 77-year-old man. The majority of secretory meningiomas have been demonstrated to cause significant perifocal edema. From a histological perspective, the inclusions and surrounding epithelial cells lead to the expression of cytokeratins, carcinoembryonic antigens, and carbohydrate antigens 19-9. In addition to the aforementioned antibodies, immunoglobulins are also less expressed, including IgA, IgG, and IgM, antitrypsin, and antichemotrypsin [4].

In this report, we present a rare histological variant of a secretory meningioma, manifested in a 77-year-old man with a clinical history of headache, impaired memory functions, partial apathetic-abulistic syndrome, and a single epileptic seizure. The surgical treatment consisted of total extirpation of the tumor, excision of the captured site on the dura, and subsequent plasty with a synthetic meningeal shell (Simpson grade I).

## Case presentation

**Figure 1:**
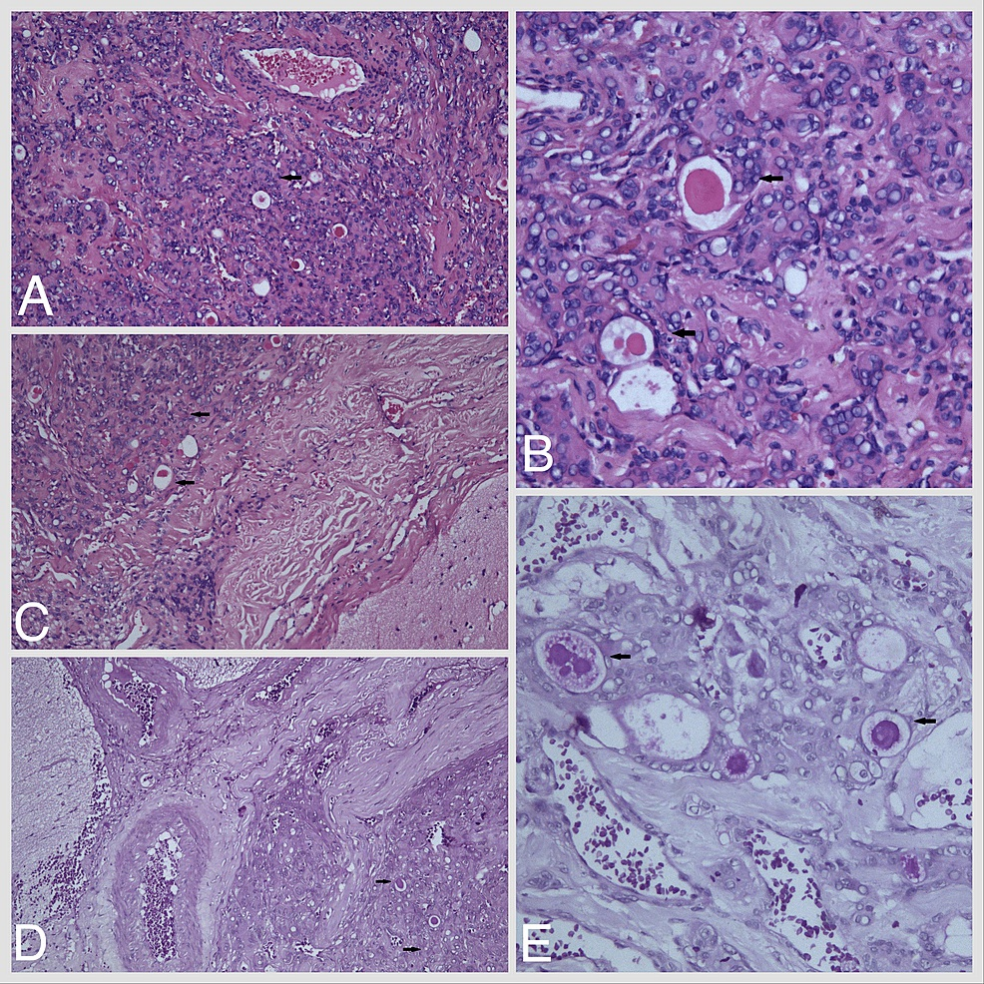
(A) Secretory meningioma with eosinophilic secretions - pseudopsammoma bodies and intracytoplasmatic eosinophilic hyaline inclusions (H.E. x 100). (B) Eosinophilic round secretions - pseudopsammoma bodies (H.E. x 200). (C) Border zone between tumor parenchyma and brain tissue (H.E. x 100). (D) Psammoma body within the tumor parenchyma (PAS x 100). (E) Foci with intracellular lumina with granular and hyalinized secretions (PAS x 200).

**Figure 2:**
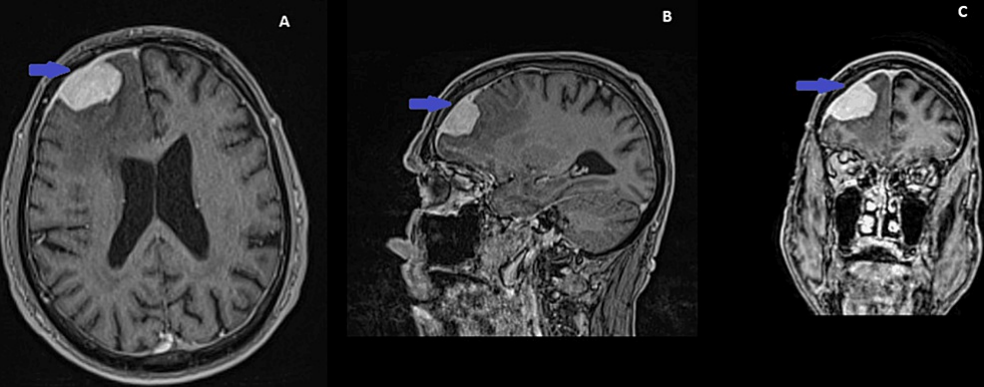
(A-C) MRI of the brain with contrast material (gadolinium) in T1 projections of axial, sagittal, and coronal sections shows a well-defined and intensely saturated hyperintense, homogeneous tumor mass located in the right frontal lobe, captured in a wide base for the convexity of the brain shell with dimensions: 2.96/1.83 cm; 2.59/2.01 cm; 3.21/2.15 cm.

A 77-year-old man presented with a history of headaches for several weeks, memory disorders, an initial form of lack of initiative and apathy, and a single epileptic seizure, on the occasion of which he was referred for a consultation with a neurosurgeon. Following a clinical examination, it was determined that the patient is clearly conscious, satisfactorily adequate, bradypsychic, and transiently uncritical. The results of the tests for latent paresis indicated the presence of a latent left hemiparesis, preserved muscle tone for the four limbs, symmetrical tendon and supraosseous reflexes, and gait instability. The patient's comorbidities include hypertension, which is being well managed without congestive heart failure, and intrahepatic bile duct carcinoma, which is currently under control. In the context of the patient's complaints regarding the central nervous system, a magnetic resonance imaging (MRI) examination of the head was conducted. A pathological formation was identified in the subdural space, with a catch site on a wide base on the confluent dural shell of the frontal lobe in the right cerebrum hemisphere. The imaging findings indicated that the tumor was consistent with a meningioma. It exhibited uniform contrast enhancement, which is indicative of a well-developed vascular supply, in the context of pronounced peritumoral edema. Histology revealed the rare variant of meningioma - secretory type (Figures 1A-1E). This latter finding is suggestive of a malignant process in the differential diagnosis. The dimensions of the axial, sagittal and coronal sections in T1-projection, with the application of intravenous contrast (gadolinium), are as follows: The dimensions of the axial, sagittal, and coronal sections in T1-projection, with the application of intravenous contrast (gadolinium), are as follows: 2.96/1.83 cm; 2.59/2.01 cm; 3.21/2.15 cm (Figures 2A-2C). The tumor was found to be non-tumorous, yet it caused marked perifocal edema (Grade 1) [5], which in turn gave rise to neurological symptoms. Consequently, the recommended treatment was total extirpation of the tumor.

**Figure 3:**
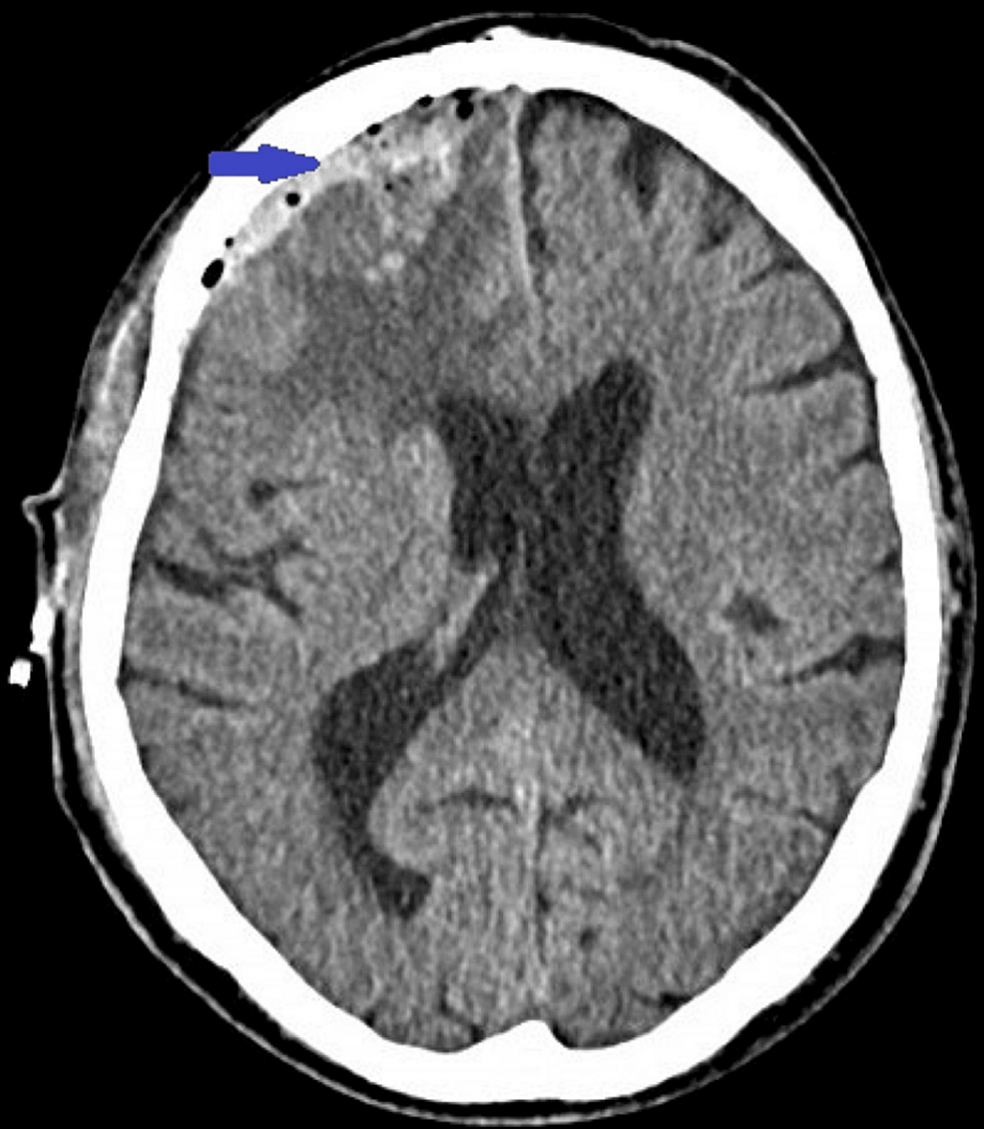
Postoperative brain CT showing a thin pellicle of subdural hemorrhagic collection (blue arrow) in the background of persistent edema of the frontal lobe of the right cerebral hemisphere.

A right frontal craniotomy was performed during the operation, during which the dura mater encephali was encountered in a tense but apparently intact state. An incision was made on the latter and immediately below it, the actual tumor formation was found. On examination, it had a granular structure and a greyish-whitish color. The tumor is a well-supplied mass, which, in a good arachnoid plan, allows for total removal (Simpson I resection was achieved) without having to sacrifice bridging venous vessels during the operative intervention. It is permissible for blood loss during the operation to be less than 200 ml. From the moment of initiation until the conclusion of the operative intervention. The total time required to perform the craniotomy, tumor removal, and subsequent tissue repair is approximately 4.5 hours. Subsequently, the patient was extubated in the intensive care unit, where he remained for a period of 48 hours for observation. The patient is clearly conscious, with no deterioration in neurological status or higher cortical functions. A follow-up computed tomography (CT) scan of the brain was performed 24 hours after the surgical intervention, which revealed the presence of a subdural sheath of blood and persistent peritumoral edema. Anti-edema and neuroprotective therapy were prescribed until the patient was discharged from the neurosurgery department (Figure 3).

The patient's treatment continued for 14 days in the hospital, after which he was monitored by means of control obstacles. This revealed a progressive improvement in his neurological status. Three months following surgical intervention, the patient exhibited clear consciousness, communication, and functionality, with no evidence of sensory or motor neurodeficiency. No further imaging studies were conducted at this stage of the clinical observation period.

## Discussion

It is a common misconception that meningiomas are benign tumors that arise from the meningothelial cells of the arachnoid and, therefore, always have adhesions to the inner surface of the dura mater encephalic. Secretory meningiomas represent a specific subgroup of benign tumors, which are classified as grade I by the WHO [3,6]. According to data from histological preparations and clinical studies conducted in the USA between 2010 and 2014, meningiomas represent 36.6% of brain tumors and 53.2% of non-malignant tumors of the CNS. The data indicate that the incidence is higher in women than in men and that with advancing age and hormonal changes, the incidence increases. Secretory meningiomas represent less than 3% of the total number of meningiomas prevalent in the CNS and are entirely benign in nature.

The microsurgical resection of convex meningiomas involves a precise and methodical dissection of the tumor from the arachnoid membrane, with a primary goal of preserving the integrity of the bridging veins to the fullest extent possible [7-9]. Unlike meningiomas of the tuberculum sellae or other critical brain regions that involve significant cranial nerves and arterial vessels, the right frontal lobe provides a relatively advantageous site for the uncomplicated surgical removal of a meningeal tumor [7]. In this instance, the tumor was entirely excised, including its attachment site on the dura. The Simpson classification, established in 1957, was utilized [8]. This classification is predicated on the understanding that meningiomas originate from dural formations, with intradural masses being the most common presentation and extradural extension occurring less frequently.

It is of significant importance to determine the histological variant of the tumor for scientific and research purposes. Secretory meningiomas are composed primarily of meningothelial cells. The tumor cells exhibit predominantly oval or round nuclei, eosinophilic cytoplasm, and indistinct cell borders, with the absence of cellular atypia. The defining feature of the secretory type of meningioma is the weak expression or rarity of psammoma bodies. Some authors define them as “pseudopsammoma bodies,” which are located intracytoplasmically. The latter are pale hyaline structures when stained with hematoxylin-eosin (H-E) [7]. A distinctive characteristic of this type of brain tumor is its positive reaction to Schiff’s acid and the presence of PAS-positive secretory granules, which serve as the primary microscopic markers for histological diagnosis [9-13]. Additionally, hyaline bodies and surrounding tumor cells exhibit immunohistochemical expression of various immunoglobulins, including IgA, IgM, and IgG. They also express alpha-1 antitrypsin, CEA, epithelial membrane antigen (EMA), and pan-cytokeratin (Pan-CK) [9-13].

Secretory meningiomas are typically associated with the presence of eosinophilic secretions, commonly referred to as pseudopsammoma bodies [6]. The most prevalent histological subtype of the tumor is meningothelial meningioma.

The histological examination revealed cells with classic meningioma histology and zones with hyalinized and eosinophilic secretions, which were identified as pseudopsammoma bodies. The aforementioned secretions are positive for periodic acid-Schiff (PAS) staining. In terms of differential diagnosis, it is important to consider the following subtypes: microcystic, clear cell, or chordoid meningioma.

A combination of KLF4 and TRAF7 genetic mutations, characteristic of secretory meningiomas, has been identified, with KLF4 mutations being unique to this type and not observed in other meningioma variants, indicating high specificity [8]. Clinical studies on the ultrastructure of secretory meningioma nuclei reveal that they are round and contain diffusely distributed euchromatin. Their cytoplasm contains a moderate number of mitochondria, a rough endoplasmic reticulum, and a Golgi apparatus [13]. According to the reviewed literature, the Ki67 proliferation index for these tumors is relatively low, typically ranging from 0% to 4% [3]. This supports the hypothesis that secretory meningiomas are of benign origin.

The perifocal edema of the brain in this type of tumor is highly pronounced, leading to radiological confusion with primary and metastatic brain tumors, an increase in intracranial volume, and the development of neurological symptoms [2,11]. Although total extirpation of the tumor renders secretory meningiomas clinically insignificant due to their benign course of development, their histological diagnosis, immunohistochemical, and genetic profile are of interest.

## Conclusions

In this clinical case, a secretory meningioma was identified in a 77-year-old male patient, a rare occurrence compared to the data reported in the literature. A significant contribution to the diagnosis is the presence of PAS-positive secretory granules, which determine the histological type of the tumor.

This case highlights the importance of the histopathological examination in accurately diagnosing, as well as a detailed description of rare tumor types, which were important for our patient. Additionally, it contributes valuable insights to the existing literature, emphasizing the significance of the rarity of the histological variant that determines the clinical approach in this patient. Given the benign nature of this type of meningioma, total tumor excision with surgical removal at the capture site is the recommended treatment, with subsequent radiotherapy not being necessary.
